# First Report of *Ophiostoma clavatum* and *Fusarium verticillioides* Associated With *Ips acuminatus*‐Infested Scots Pine in Western Ukraine

**DOI:** 10.1002/pei3.70134

**Published:** 2026-02-23

**Authors:** Yurii Yusypovych, Yuliia Shalovylo, Oleh Kit, Volodymyr Kramarets, Volodymyr Zaika, Mykola Korol, Vasyl Lavnyy, Hryhorii Krynytskyi, Valentina Kovaleva

**Affiliations:** ^1^ Ukrainian National Forestry University Lviv Ukraine; ^2^ Sudova Vyshnya Lyceum Named After Tadei Dmytrasevych Lviv Ukraine; ^3^ Department of Agricultural Chemistry and Soil Science Vasyl Stefanyk Carpathian National University Ivano‐Frankivsk Ukraine; ^4^ Department of Regulation of Cell Proliferation and Apoptosis Institute of Cell Biology of NASU Lviv Ukraine

**Keywords:** antagonism, blue‐stain fungi, *Fusarium*, pathogenicity, pine engraver beetle, *Pinus sylvestris* L.

## Abstract

Over the past two decades, dieback of *Pinus sylvestris* L. stands has increased across Europe, largely due to mass outbreaks of the bark beetle, in particular, *Ips acuminatus* Gyll. (Coleoptera: Curculionidae). This beetle causes mechanical damage and vectors pathogenic fungi, including ophiostomatoid species that induce blue stain. *Ophiostoma clavatum* Math.‐Käärik is the most frequently reported fungal associate, yet its occurrence has not been documented in Ukraine. While ophiostomatoid fungi are well studied in pine pathogenesis, the role of fast‐growing co‐occurring associates such as *Fusarium* spp. remains poorly understood. This study aimed to identify the dominant *Ophiostoma* and *Fusarium* species associated with *I. acuminatus* in western Ukraine and to evaluate their pathogenicity and in vitro interactions. Isolates from surface‐sterilized beetle abdomens and blue‐stained wood were identified as *O. clavatum* based on morphology and multi‐locus molecular markers (ITS, TUB, TEF1‐α). Pathogenicity tests showed that *O. clavatum* acts as a weak phytopathogen, primarily inducing localized lesions. The dominant *Fusarium* morphotype from blue‐stained wood was identified as *Fusarium verticillioides* (Sac) Nirenberg, which induced severe necrosis and tissue maceration on pine seedlings. In dual culture, *F. verticillioides* displayed strong asymmetric competitive dominance over *O. clavatum*, reducing its growth by more than 45%. This study provides the first record of *O. clavatum* associated with *I. acuminatus* in Ukraine, extending its known European distribution. The observed pathogenicity and competitive ability of *F. verticillioides* suggest it may synergistically contribute to Scots pine decline, warranting further investigation into its role within the beetle–fungus complex.

## Graphical abstract

This study provides the first record of *Ophiostoma clavatum* associated with *Ips acuminatus* in Ukraine, and the first evidence of *Fusarium verticillioides* coexisting with ophiostomatoid fungi in infested Scots pines. This fungal association may contribute to the decline of Scots pine stands.

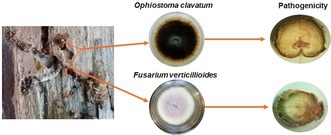

## 1 Introduction

Scots pine ( *Pinus sylvestris* L.) is a widely distributed forest species of major ecological and economic importance. It is highly adaptable, thriving on nutrient‐poor soils and tolerating extreme conditions. However, climate change‐induced fluctuations impair its photosynthetic activity and weaken its defenses against stress factors such as bark beetle attacks (Dobbertin et al. 2007; Brichta et al. 2024).

*Ips* species, traditionally secondary pests that attack weakened or damaged pines, have recently increased in impact due to extreme weather events such as droughts and heatwaves. These conditions reduce forest resilience and alter pest dynamics (Wermelinger et al. 2008; Siitonen 2014). Rising pine damage linked to *Ips acuminatus* Gyll. (Coleoptera: Curculionidae) has been reported across Europe, including France, Switzerland, Italy, Finland, Ukraine, Czechia, and Poland (Lieutier et al. 1991; Dobbertin et al. 2007; Wermelinger et al. 2008; Colombari et al. 2012, 2013; Siitonen 2014; Davydenko et al. 2017; Knížek and Liška 2023; Jankowiak et al. 2023; Yusypovych et al. 2023). Bark beetles form complex associations with fungi, particularly ophiostomatoid species (blue‐stain fungi), which discolor colonized sapwood (Wingfield and Van Wyk 1993; Kirisits 2004; Linnakoski et al. 2012). Ophiostomatoid fungi weaken hosts by disrupting water transport, metabolizing terpenes, and altering tree chemistry, thereby helping beetles overcome tree defense system and accelerating tree death (Guérard et al. 2007; Zaman et al. 2023). In return, fungi enhance nitrogen availability and modify phloem compounds to benefit brood establishment (Lieutier et al. 2009; Villari et al. 2012). Ecologically, ophiostomatoid fungi play a dual role: on the one hand, they promote outbreaks of bark beetles that cause large‐scale forest disturbances; on the other hand, they contribute to wood decomposition and nutrient cycling which, in turn, enriches forest ecosystems, helps maintain soil fertility, and fosters the development of resilient forest communities. (Siegert et al. 2024).

As detailed by de Beer et al. (2022), ophiostomatoid fungi represent a morphologically convergent but phylogenetically disparate group. *I. acuminatus* is closely linked to fungi from diverse genera including *Ceratocystiopsis* (Mathiesen 1950; Lieutier et al. 1991), *Graphilbum* (Waalberg 2015; Davydenko et al. 2017; Jankowiak et al. 2023), *Grosmannia* (Davydenko and Baturkin 2020), *Leptographium* (Mathiesen‐Käärik 1953; Jankowiak et al. 2023), *Ophiostoma* (Mathiesen 1951; Francke‐Grosmann 1952; Linnakoski et al. 2012, 2016; Villari et al. 2012; Davydenko et al. 2017), and Sporothrix (Jankowiak et al. 2023) in the order Ophiostomatales as well as *Endoconidiophora* (Mathiesen 1950; Francke‐Grosmann 1952) and *Graphium* (Waalberg 2015; Davydenko and Baturkin 2020; Jankowiak et al. 2023) from Microascales.

The most frequently reported associate of *I. acuminatus* across multiple studies in several European countries is *Ophiostoma clavatum* Math.‐Käärik (Mathiesen 1950, 1951; Rennerfelt 1950; Mathiesen‐Käärik 1953; Francke‐Grosmann 1963; Käärik 1975; Lieutier et al. 1991; Guérard et al. 2000; Villari et al. 2012; Linnakoski et al. 2016). Since 2010, drought and outbreaks of the aggressive bark beetles *I. acuminatus* and *I. sexdentatus* have caused extensive dieback of *P. sylvestris* in Ukraine (Davydenko et al. 2017, 2021), affecting some 79,000 ha of pine stands in 2022. Remarkably, the *O. clavatum* species complex, typically dominant in association with the pine engraver beetle in Western and Northern Europe, was not detected in recent studies in Eastern Ukraine, where the species present belonged to the *O. ips*, *O. minus*, and *O. piceae* complexes (Davydenko et al. 2017; de Beer et al. 2022). These regional variations prompted us to identify the dominant *Ophiostoma* species associated with *I. acuminatus* in the western region of Ukraine, which is characterized by higher humidity and less pronounced temperature fluctuations than the eastern regions (the distance between these areas is approximately 1000 km).

In addition to ophiostomatoid fungi, pine bark beetles also carry other aggressive pathogens. *Sphaeropsis sapinea* (Fr.) Dyko & B. Sutton has been reported at high frequency in association with *I. acuminatus* (Davydenko et al. 2017), while *Fusarium circinatum* Nirenberg & O'Donnell has been detected in galleries of *I. sexdentatus* (Bezos et al. 2018). Co‐infections with such pathogenic fungi and ophiostomatoid fungi may further exacerbate tree decline (Elvira‐Recuenco et al. 2020). Notably, *Fusarium* species comprised 3.17% of the *I. acuminatus* microbiome (Chakraborty et al. 2020), suggesting this beetle may vector fusaria.

The objectives of this study were to: (1) identify the dominant ophiostomatoid morphotypes associated with *I. acuminatus* in Lviv region (western Ukraine); (2) isolate and characterize the dominant fast‐growing fungi, specifically *Fusarium* spp., associated with blue‐stain sapwood; (3) assess the pathogenicity of selected *Ophiostoma* and *Fusarium* isolates on Scots pine seedlings, and (4) evaluate the in vitro interactions between dominant *Ophiostoma*‐like and *Fusarium* isolates to understand their competitive or synergistic dynamics.

## 2 Materials and Methods

### 2.1 Study Site and Sampling

This study was conducted in August 2022 in a 50–60‐year‐old pure stand of Scots pine (total area 2.3 ha) located in the “Rava‐Ruska Forestry” (50°23′ N, 23°66′ E) of the Lviv Region, Ukraine. Since 2015, two to three outbreak foci of Scots pine dieback have occurred annually in this pine stand, caused by mass colonization of the trees by the pine engraver beetle *I. acuminatus* , despite the annual removal of infested pines from the stand (Kramarets et al. 2023). In an active medium‐sized spot of the bark beetle infesting Scots pine (Colombari et al. 2013), five living pine trees, located in different parts of the spot (with distance between them ranging from 50 to 150 m), were selected. Sampled trees showed visible infestation symptoms including needle discoloration, crown thinning (> 40%), and beetle entry holes. These trees were felled, and logs containing beetle galleries from the upper stem sections and branches were collected and transported to the laboratory on the same day (Figure S1).

### 2.2 Culture‐Based Isolation

In the laboratory, 20 *I. acuminatus* beetles per tree were collected from galleries using fine forceps, resulting in a total sample size of 100 beetles. Insect samples were rinsed with sterile distilled water, surface‐sterilized in 96% ethanol for 3 min, and then rinsed twice with sterile water. The efficacy of the sterilization was verified by plating the final wash onto 2% potato dextrose agar (PDA) and incubating at 24°C for 5 days. Head‐pronota with wings and legs were aseptically removed. Abdominal segments of beetles from each tree were pooled and transferred to 2 mL microcentrifuge tubes containing 10 mM sterile phosphate‐buffered saline. This surface‐sterilization and dissection protocol was employed to specifically target internal microbial associates while minimizing environmental contaminants. The samples were homogenized on ice using a plastic pestle, followed by vortexing for 1 min at 2400 rpm (Biosan, Latvia). The homogenates were centrifuged at 4000 rpm for 5 min to separate the microbial suspension from insect tissue debris (Hu et al. 2015). Supernatants were plated onto 2% PDA supplemented with antibiotics (100 μg/mL ampicillin and 50 μg/mL chloramphenicol), and incubated at 24°C in the dark. All procedures were carried out under sterile conditions using a microbiological safety cabinet (Walker, Glossop, UK). Petri dishes were examined daily for 4 weeks. Slowly growing colonies with dark‐brown to black pigmentation were subcultured in Petri dishes with PDA supplemented with 0.15% malt extract (MPDA) with antibiotics.

Forty blue‐stained wood samples (5 × 5 mm^2^), collected adjacent to galleries (eight samples per tree), were surface‐sterilized in 70% ethanol for 1 min. The samples were then rinsed three times with sterile distilled water, dried on sterile filter paper, and placed on PDA amended with antibiotics. The plates were incubated at 24°C for 3 weeks. Colonies were selectively subcultured based on growth rate and morphology to prioritize dominant taxa: fast‐growing isolates (> 1 cm/day) with cottony aerial mycelium were selected as *Fusarium* candidates, while slow‐growing, dark‐brown to black colonies were targeted for *Ophiostoma‐*like. All selected isolates were purified on MPDA and grouped into fungal morphotypes (FMs) based on macroscopic and microscopic characteristics observed using a Jenaval stereomicroscope (Carl Zeiss, Jena).

### 2.3 Molecular Identification

The representative isolate F3‐1 of the *Fusarium*‐like dominant fungal morphotype (DFM), comprising seven isolates from blue‐stained wood, and isolate B‐0922 of the *Ophiostoma*‐like DFM, consisting of 13 isolates from trees and insects, were subjected to molecular identification. Genomic DNA was extracted using the GeneJET Plant Genomic DNA Purification Kit (Thermo Fisher Scientific). The ITS region was amplified for both isolates using primers ITS1F/ITS4R (Gardes and Bruns 1993; White et al. 1990). For the *Ophiostoma*‐like isolate B‐0922, the β‐tubulin (*β‐TUB*) gene was amplified using primers T10/Bt2b (O'Donnell and Cigelnik 1997; Glass and Donaldson 1995), and the translation elongation factor 1α (*TEF1‐α*) gene region with primers EF1F/EF2R following the protocols of Jacobs et al. (2004). For identification of isolate F3‐1, in addition to the ITS region, three loci were amplified: TEF1‐α using EF1/EF2 primers (O'Donnell et al. 1998); the DNA‐directed RNA polymerase II largest subunit (RPB1) with Fa/G2R primers (Hofstetter et al. 2007; O'Donnell et al. 2010); and the second largest subunit (RPB2) using 5F2/7Cr primers (Reeb et al. 2004; Liu et al. 1999), following protocols described by Yilmaz et al. (2021). PCR products were purified with QIAquick PCR Purification Kit (Qiagen, Hilden, Germany). Sequencing was carried out by Explogen LLC (EXG, Lviv, Ukraine). Sequences were compared to GenBank via BLASTn, with identification criteria set at > 80% coverage, 98%–100% similarity for species, and 94%–97% for genus. New sequences were deposited in NCBI GenBank.

### 2.4 Phylogenetic Analysis

A multilocus sequence analysis (MLSA) of isolate F3‐1 was conducted using partial sequences of the *TEF1‐α* (GenBank accession PQ527913.1), *RPB1* (PV976806.1), and *RPB2* (PV976805.1) genes. Species identification was supported by comparing these sequences against the FUSARIOID‐ID database (Geiser et al. 2004). The top matches were further validated using BLAST on the NCBI platform (http://blast.ncbi.nlm.nih.gov) to assess similarity and infer phylogenetic relationships (Table S1).

Multiple sequence alignments for each locus were generated with the ClustalW algorithm in MEGA 11. The MLSA was performed using MEGA X (v. 11) to construct a Maximum Likelihood (ML) phylogeny based on the Kimura 2‐parameter (K2P) model, with branch support evaluated by 1000 bootstrap replicates (Tamura et al. 2021).

### 2.5 Pathogenicity Tests

Pathogenicity of fungal isolates was assessed on Scots pine seedlings grown in sandy loam soil under natural irrigation at the Botanical Garden of the National Forestry University of Ukraine (Lviv, Ukraine). To evaluate host response and vitality, metrics included lesion dimensions, resin exudation, and the capacity for wound healing.

For inoculum preparation, *O. clavatum* isolate B‐0922 was cultured on 2% malt‐extract agar (MEA) at 24°C for 25 days. Agar blocks with fungal mycelium (ca. 5 mm in diameter) were aseptically excised. In May 2023, 15 three‐year‐old seedlings were inoculated: bark at 10–12 cm above the root collar was sterilized with 70% ethanol, a 1.5 × 0.6 cm incision was made to the sapwood, and an agar block with actively growing mycelium was inserted beneath the bark flap, facing the wound. The site was sealed with Parafilm. Controls (15 seedlings) received sterile agar. Seedlings were monitored weekly for 21 weeks, then harvested. Lesion length and depth were measured, and re‐isolations were performed from lesion margins on MEA. Fungal identity was confirmed by micromorphology.

Pathogenicity of *Fusarium verticillioides* isolate F3‐1 was tested on both 7‐day‐old and 2‐year‐old seedlings. Seedlings germinated from sterilized seeds were placed on sterile filter paper over 1% water agar in Petri dishes (10 seedlings per treatment). Root zones were inoculated with a spore suspension (7 × 10^5^ CFU/mL) from a 10‐day‐old PDA culture; sterile water was used as a control. After 9 days, seedling health was assessed under a stereomicroscope. Experiments were run in triplicate.

In July 2023, 15 two‐year‐old seedlings were inoculated with F3‐1. Sites were surface‐sterilized as above, and a bark flap was cut to the sapwood. A 2 × 2 mm agar block with actively growing mycelium from a 10‐day‐old culture was inserted, the flap replaced, and the wound sealed with Parafilm. Controls (15 seedlings) received sterile agar. Seedlings were monitored weekly for 8 weeks, then harvested. Host vitality was assessed by monitoring resin flow and the extent of cambial wound closure. Necrotic tissue fragments were collected for re‐isolation of inoculated fungi.

### 2.6 Antagonism Assay

To investigate the interactions between *F. verticillioides* F3‐1 and *O. clavatum* В‐0922, a dual‐culture assay was conducted. Mycelial plugs (5 mm in diameter) were taken from each fungal culture and placed 3 cm apart on the surface of MPDA.

In the control group, mycelial plugs of each fungus were placed individually at the center of separate MPDA plates. Petri dishes were incubated in the dark at 24°C. Fungal growth was assessed 3 and 7 days after inoculation. Interactions were evaluated by observing colony inhibition, directional growth, and pigmentation at the contact zone. All treatments and controls were performed in triplicate.

### 2.7 Statistical Analysis

Data are presented as means ± standard errors. The results of the inoculation test were analyzed using a one‐way analysis of variance (ANOVA), followed by Dunnett's test (*p* ≤ 0.05) to compare each fungal isolate with the control. All analyses were performed using XLSTAT software.

## 3 Results

### 3.1 Morphological and Molecular Identification of Fungal Isolates

**FIGURE 1:**
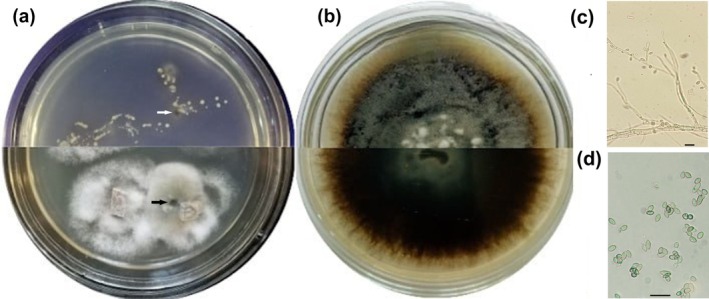
Morphological characteristics of *Ophiostoma clavatum*. (a) Isolates obtained from abdominal segments of *I. acuminatus* (top of plate) and blue‐stained sapwood of *P. sylvestris* (bottom); (b) three‐week‐old culture on PDA supplemented with 0.15% malt extract (surface and reverse); (c) hyalorhinocladiella‐like asexual morph; (d) conidia. Arrows indicate *Ophiostoma*‐like colonies. Scale bars: 10 μm.

A total of 26 Ophiostoma‐like isolates were obtained, half of which were derived from insect abdomens and the other half were from blue‐stained sapwood fragments (Figure 1a). Based on colony morphology, these isolates were grouped into four distinct FMs, representing a phylogenetically diverse assemblage within the sampled galleries. The DFM comprised 13 isolates (7 from bark beetles and 6 from sapwood). The second most abundant FM consisted of eight isolates (5 from insects and 3 from sapwood), while the remaining two FMs included two and three isolates, respectively, all obtained from sapwood.

The DFM produced deep dark‐brown mycelium with superficial white‐beige aerial hyphae and smooth, hyaline margins on MPDA (Figure 1b), with an average radial growth rate of 4.1 ± 0.6 mm/day at 24°C. Conidiophores typical of the genus *Ophiostoma* were observed, forming small, brush‐like clusters (Linnakoski et al. 2016). These structures consisted of thin, elongated, septate hyphae that were often branched, bearing short lateral branches. Slight swellings indicated the positions of conidiogenous cells. Phialides were small, cylindrical to slightly clavate, and usually clustered. Conidia were unicellular, 3–4 μm long, oval to ellipsoidal, and borne singly or in short chains (Figure 1c,d). Isolate B‐0922, a representative of this DFM, was selected for molecular identification.

The ITS sequence of isolate B‐0922 (GenBank accession OR799511.1) showed the highest similarity to *O. clavatum* and *O. brunneolum* (Table 1). Although ITS region does not fully resolve closely related taxa, it confirmed affiliation with the *O. clavatum* complex (Linnakoski et al. 2016). For species‐level resolution, partial β‐TUB and TEF1‐α sequences (accessions PX237223.1, PX237224.1) were analyzed, both showed the highest identity (100% and 99.72%, respectively) to *O. clavatum* strain CMW37983. Combined morphological and molecular evidence confirmed that isolate B‐0922, obtained from the abdomen of *I. acuminatus* , belongs to *O. clavatum*. Preliminary examination of isolates from the second most abundant FM also showed their affiliation with the *O. clavatum* complex (GenBank accession OR799512.1); however, significant sequence divergence suggests these isolates may represent a putative new species within the complex, which is currently under further investigation.

**TABLE 1:** Molecular identification of isolate B‐0922.

Isolate	GenBank accession no.	Origin	Gene region	% sequence coverage	% identity
*O. clavatum* isolate CMW37983	OM501477.1	Sweden	ITS	90	100
*O. clavatum* isolate CMW37983	KU094685.1	Sweden	ITS	93	99.83
*O. brunneolum* isolate CMW44477	MH144077.1	China	ITS	100	99.51
*O. hongxingense* strain HXS_66	MK748194.1	China	ITS	100	99.06
*O. clavatum* isolate CMW37983	KU094705.1	Sweden	β‐TUB	100	100
*O. clavatum* isolate CMW41041	KU094710.1	Sweden	β‐TUB	100	99.69
*O. piliferi* (nom. inval.) strain CXY4047	OM678560.1	China	β‐TUB	100	96.05
*O. brunneolum* isolate CMW44477	MH124272.1	China	β‐TUB	100	95.17
*O. clavatum* isolate CMW37983	KU094759.1	Sweden	TEF1‐α	85	99.72
*O. clavatum isolate* CMW41043	KU094763.1	Norway	TEF1‐α	85	98.74
*O. brunneolum* isolate CMW44477	MH124357.1	China	TEF1‐α	93	94.46

**FIGURE 2:**
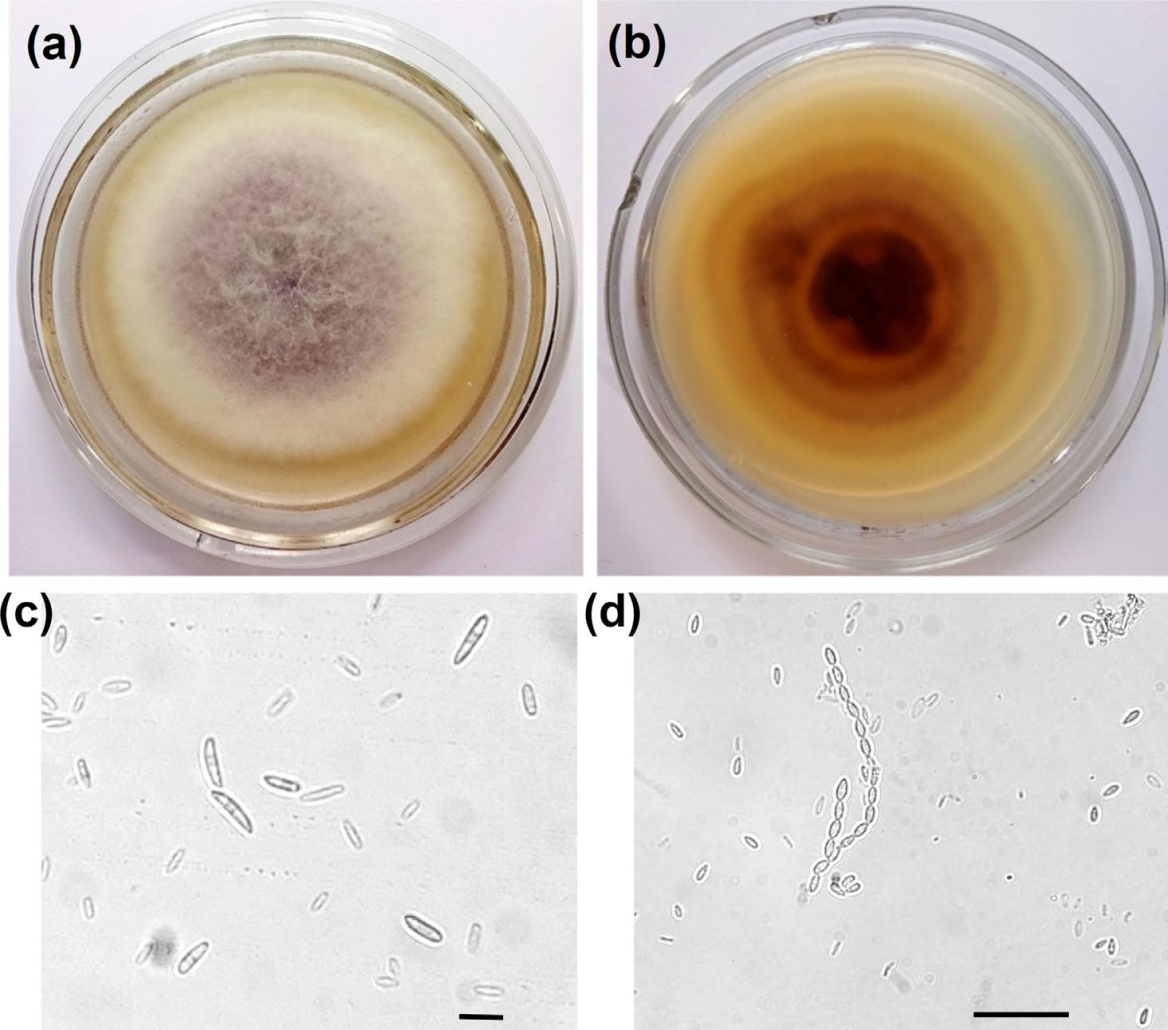
Morphological characteristics of *Fusarium verticillioides*. (a, b) Seven‐day‐old culture on PDA supplemented with 0.15% malt extract (surface and reverse); (c) macroconidia; (d) microconidia. Scale bars: 20 μm.

*Fusarium*‐like fungi were detected in 45% of the blue‐stain sapwood samples, yielding 18 isolates that were grouped into six FMs. One dominant type, comprising seven isolates from four trees, was selected for further study. On MPDA after 7 days at 24°C, colonies appeared cottony to floccose, with a white to cream margin and violet‐gray center (Figure 2a). The reverse side exhibited yellow to dark‐brown pigmentation with concentric rings (Figure 2b). Radial growth averaged 15.1 ± 1.8 mm/day. Macroconidia were slightly falcate, predominantly 3‐septate, and 20–40 μm long (Figure 2c). Microconidia were unicellular, hyaline, oval to club‐shaped, 5–8 μm long, and produced in chains or false heads (Figure 2d), characteristics typical of the *Fusarium fujikuroi* species complex (FFSC) (Bao et al. 2023).

ITS sequencing of representative isolate F3‐1 (GenBank accession PQ488808.1) revealed 99.64% identity (98% coverage) to *Fusarium cf. fujikuroi* isolate 56339C (PP804370.1) and *F. verticillioides* isolate BFO (PP434657.1), and 99.45% identity to *F. circinatum* , *F. coicis*, *F. temperatum*, and *F. napiforme*—all members of the FFSC.

**FIGURE 3:**
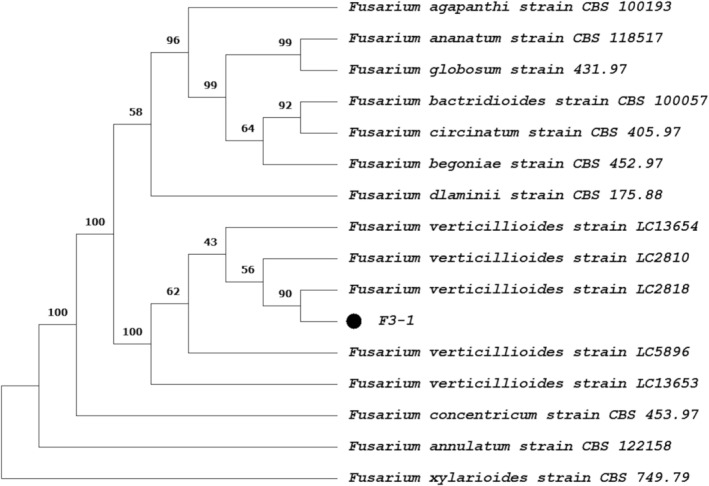
Maximum likelihood (ML) tree of *F. verticillioides* isolate F3‐1 based on a combined three‐gene dataset (*tef1‐α*, *rpb1*, and *rpb2*) generated in MEGA 11.0. Values above nodes represent bootstrap support (1000 replicates).

A ML phylogenetic tree based on the combined partial sequences of tef1‐α, rpb1, and rpb2 clearly resolved two major clades with strong statistical support (bootstrap = 100). The first clade included diverse *Fusarium* species (*F. agapanthi*, *F. ananatum*, *F. globosum*, *F. bactridioides*, *F. circinatum*, *F. begoniae*, and *F. dlaminii*), while the second comprised representatives of *F. verticillioides* along with *F. concentricum*, *F. annulatum*, and *F. xylarioides*. Isolate F3‐1 clustered within the *F. verticillioides* clade, most closely to strain LC2818 (bootstrap support 90%), confirming its identification as *F. verticillioides* (Figure 3).

### 3.2 Pathogenicity Tests

**FIGURE 4:**
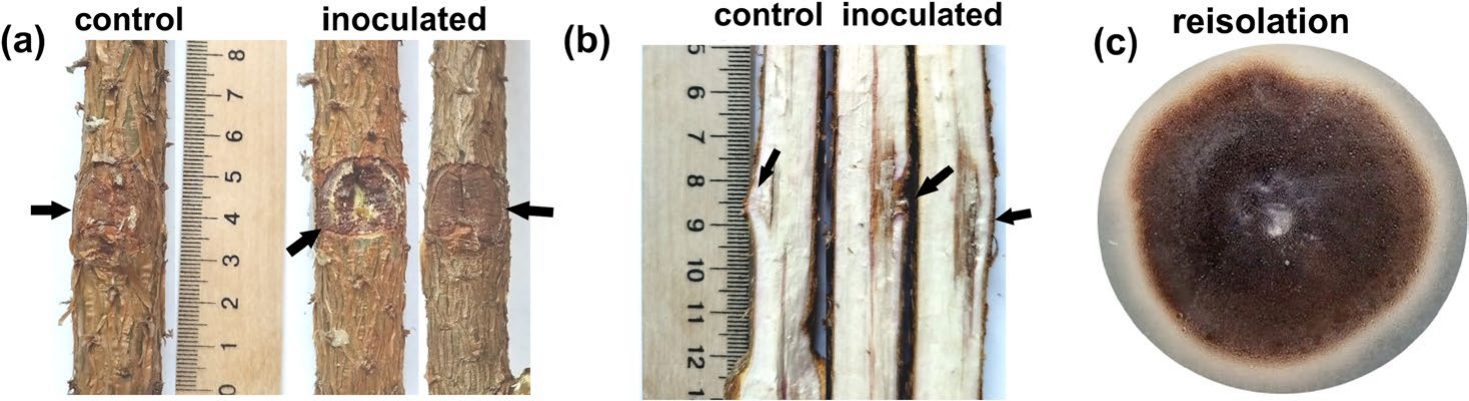
Pathogenicity of *O. clavatum*. Three‐year‐old *P. sylvestris* seedlings were inoculated beneath bark flaps with agar blocks containing *O. clavatum* mycelium (inoculated) or sterile agar (control). Symptoms after 21 weeks: (a) inoculation site on stem; (b) longitudinal stem section through the inoculation site; (c) culture reisolated from inoculated seedlings. Arrows indicate inoculation points.

Twenty‐one weeks after inoculation, 60% of *P. sylvestris* seedlings inoculated with *O. clavatum* developed moderate bleeding lesions on the outer bark (Figure 4a), while the remaining seedlings showed only slight resin exudation. Control seedlings exhibited dry wounds with only localized discoloration. Inoculated plants developed elongated dark‐brown lesions extending vertically from the inoculation site (Figure 4b). Lesion length and depth in controls averaged 15.2 ± 1.3 and 1.2 ± 0.1 mm, respectively, compared with 30.5 ± 3.6 and 4.4 ± 0.6 mm in inoculated seedlings. These differences were statistically significant (*p* < 0.001). The inoculated fungus was successfully reisolated from 100% of inoculated seedlings and matched the original isolate morphologically (Figure 4c). No fungi were recovered from control plants.

**FIGURE 5:**
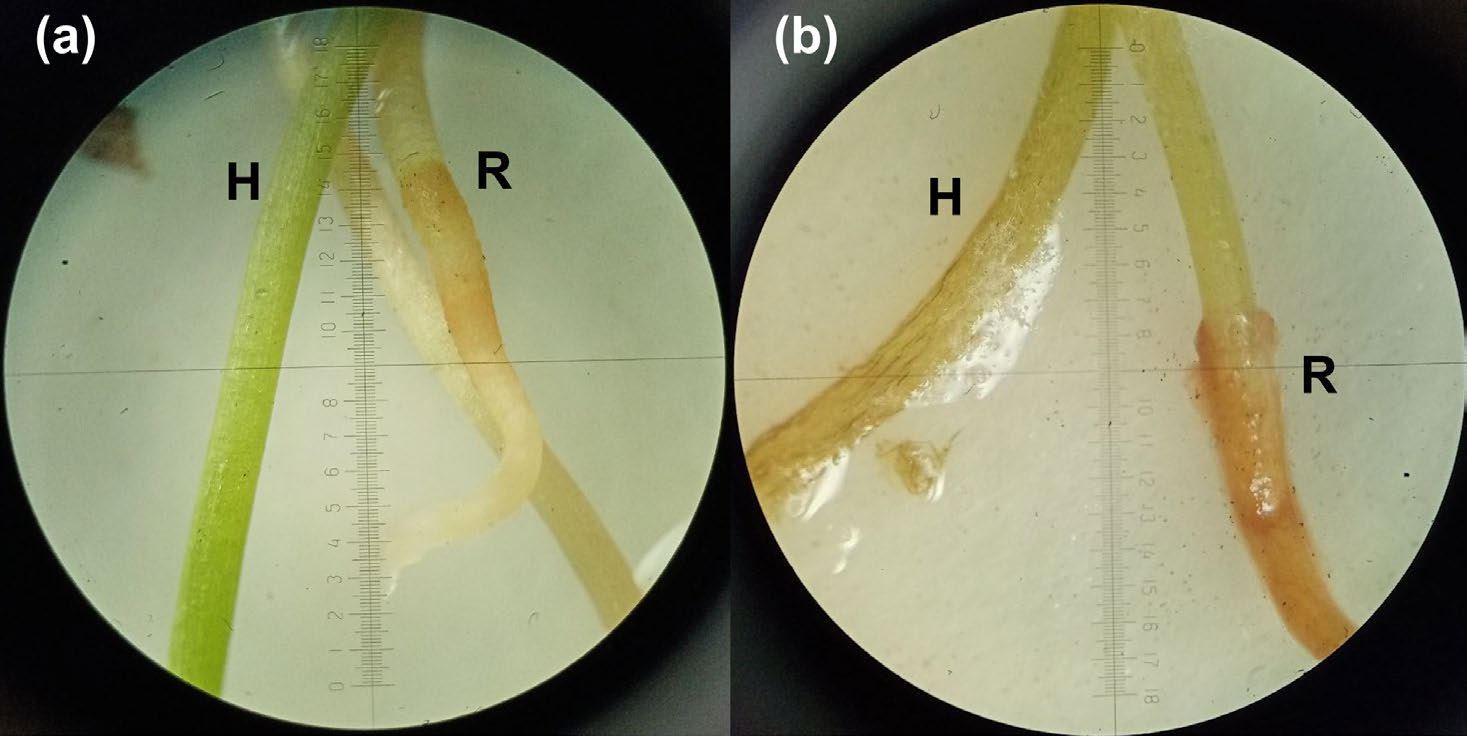
Pathogenicity test of *F. verticillioides* on seven‐day‐old *P. sylvestris* seedlings. (a) Control seedlings showed no pathological changes; (b) inoculated seedlings displayed root and hypocotyl browning, white aerial mycelium on hypocotyls, and tissue maceration. H, hypocotyl; R, root.

Inoculation of seven‐day‐old seedlings with *F. verticillioides* caused disease symptoms in 84.3% ± 4.6% of plants, including root and hypocotyl browning, white aerial mycelium on hypocotyls, black necrotic strands along vascular tissues, tissue maceration, and cotyledon damage. Control seedlings remained symptomless (Figure 5).

**FIGURE 6:**
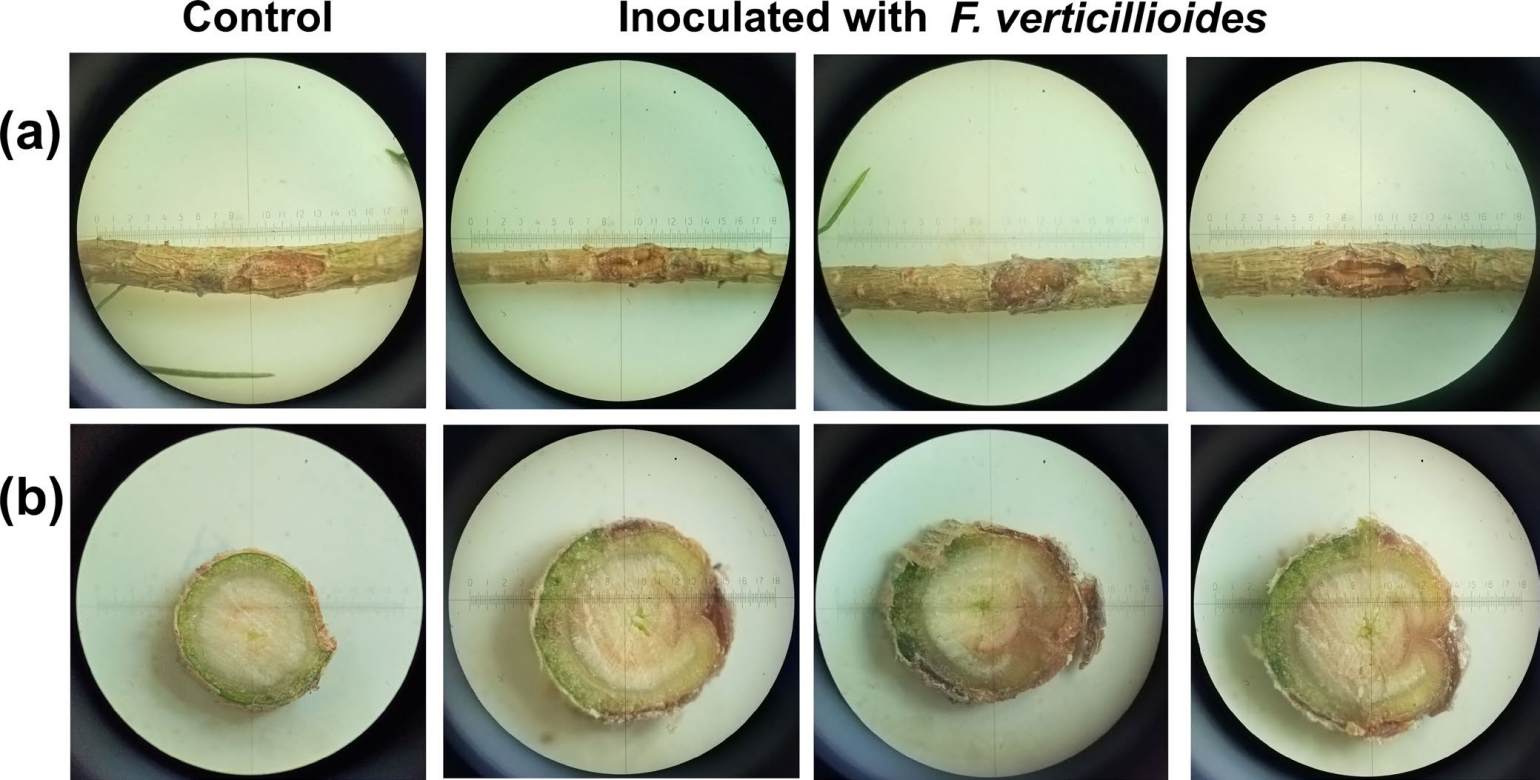
Pathogenicity test of *F. verticillioides* on two‐year‐old seedlings. Representative symptoms after 8 weeks: (a) at inoculation site on stem; (b) cross‐section through inoculation site.

In 2‐year‐old seedlings inoculated beneath bark flaps, all plants developed disease symptoms. A critical assessment of tree vitality revealed that while control seedlings showed complete wound closure via cambial growth, *F. verticillioides*‐inoculated plants exhibited unhealed wounds and persistent resin exudation (73.3% of seedlings; Figure 6a). Necrotic lesions extended 5.8 ± 0.8 mm from the site, and wood discoloration reached the second annual ring in 60.4% of seedlings, indicating deep tissue penetration (Figure 6b). The pathogen was reisolated from all inoculated plants.

### 3.3 Antagonism Assay

**FIGURE 7:**
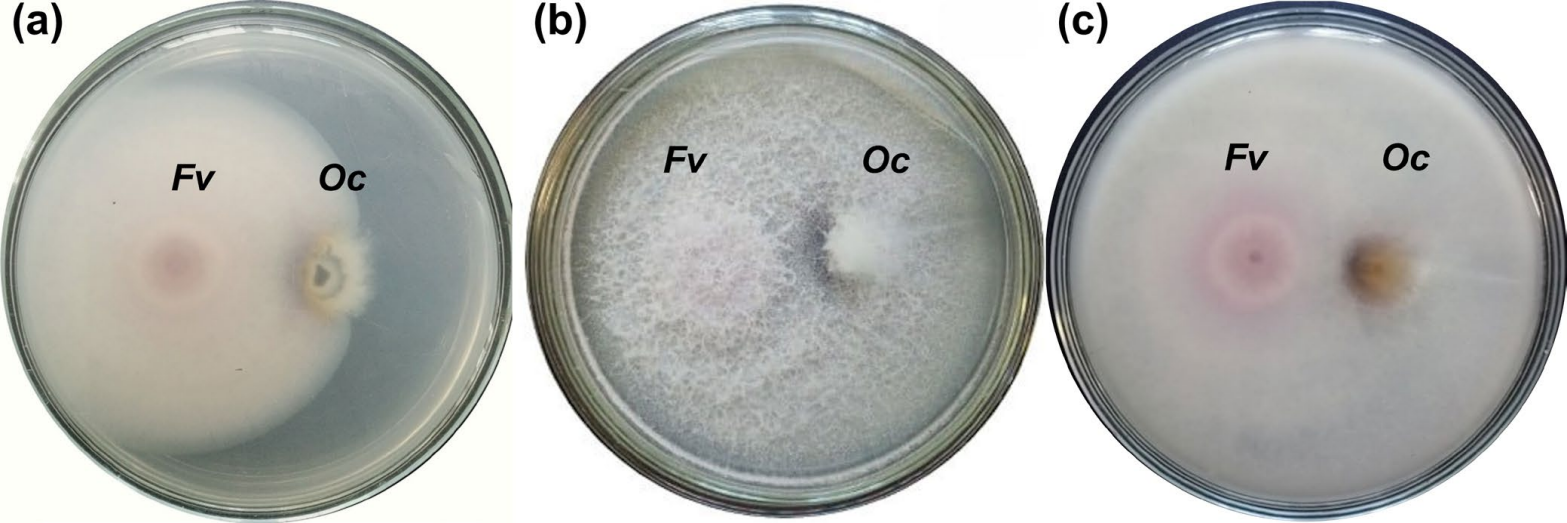
Dual‐culture assay of *F. verticillioides* F3‐1 (*Fv*) and *O. clavatum* B‐0922 (*Oc*) on PDA supplemented with 0.15% malt extract plates. (a) Reverse view after 3 days; (b) front view after 7 days; (c) reverse view after 7 days.

After 3 days in dual culture, *F. verticillioides* exhibited rapid mycelial expansion with diffuse pink pigmentation at the colony center. Colonies of *O. clavatum* showed a denser structure and directional growth away from the co‐inoculant, suggesting a negative chemotropic response (Figure 7a). The average colony diameter was 14.1 ± 1.2 mm, comparable to the control. By day seven, *F. verticillioides* had colonized most of the plate, producing dense, cottony white aerial mycelium on the surface and distinct pink pigmentation on the reverse. In contrast, *O. clavatum* formed smaller, compact colonies with dense aerial mycelium and a dark‐brown zone at the contact area (Figure 7b,c). Colony diameter of *O. clavatum* in dual culture was reduced to 16.1 ± 1.2 mm compared to 29.4 ± 1.6 mm in the control, confirming the strong suppressive effect of *F. verticillioides* on the growth of the ophiostomatoid partner.

## 4 Discussion

This study provides the first report of *O. clavatum* associated with *Ips acuminatus* in the Scots pine forests of western Ukraine. Although the sample size (100 beetles and 40 wood samples from five trees) is relatively modest, it proved sufficient to consistently detect and identify the dominant fungal associate across both insect and wood substrates within this localized outbreak focus. Similar sample sizes have previously been shown to be adequate for characterizing primary blue‐stain fungi vectored by *I. acuminatus* in the Southern Alps (Villari et al. 2013). This widespread beetle–fungus association has been documented across Austria, France, Germany, Norway, Italy, Sweden, and the former Yugoslavia (Francke‐Grosmann 1952, 1963; Lieutier et al. 1991; Villari et al. 2013; Linnakoski et al. 2016). Based on these data, it has been suggested that this fungus is a specific associate of the bark beetle, showing limited spatial variation (Villari et al. 2012; Linnakoski et al. 2016). Our study area, located approximately 1000 km from the nearest previously reported localities for this complex, further supports the assumption of broad geographic consistency.

However, recent investigations of fungal communities associated with *I. acuminatus* on Scots pine in a Polish region approximately 400 km from our study site, characterized by comparable climatic conditions (Jankowiak et al. 2023), as well as in eastern Ukraine, where the growing season features high temperatures and low humidity (Davydenko et al. 2017), failed to detect *O. clavatum*. These findings indicate significant regional variation in fungal assemblage composition, which may be influenced by climatic factors or methodological differences. A primary source of such discrepancies in reported fungal occurrence and frequency lies in differences between sampling, isolation, detection and identification protocols (Kirisits 2004; Linnakoski et al. 2016; Papek et al. 2024).

In the present study, we adopted a culture‐based approach incorporating surface sterilization to filter out “environmental noise” from fast‐growing opportunistic saprotrophs. The beetle exoskeleton and galleries are open environments that accumulate transient spores from various sources (e.g., trees, mites, other insects), often harboring a taxonomically diverse fungal community (Davydenko et al. 2017). Due to interspecific antagonism, slow‐growing ophiostomatoids can be overlooked if their growth is suppressed by faster‐growing microorganisms on culture media (Furniss et al. 1990). Our use of surface sterilization specifically targeted internal or strongly attached associates. Given that *I. acuminatus* is a phloeomycetophagous species—feeding on both phloem and fungal structures (Francke‐Grosmann 1952)—and that the insect digestive tract typically hosts a more consistent “core mycobiome” (Hu et al. 2015; Chakraborty et al. 2020), we selected the isolation from beetle abdomens as a reliable method to identify stable symbionts. The consistent recovery of *O. clavatum* and a second, potentially new species of the same complex from surface‐sterilized beetles and adjacent blue‐stained sapwood provides strong evidence of robust association in this region.

Previous inoculation experiments have demonstrated that *O. clavatum* was shown to be phytopathogenic but exhibits only low virulence toward its host (Guérard et al. 2000; Villari et al. 2012; Isberg et al. 2022). Pathogenicity is a key factor that influences both the outcome of fungal competition during colonization of living sapwood and the ability of fungi to overcome tree defenses (Six and Wingfield 2011). Pathogenicity among ophiostomatoid fungi varies widely, ranging from non‐pathogenic or weakly pathogenic species to highly virulent ones capable of killing healthy trees; moreover, pathogenicity can differ substantially even among isolates of the same species (Linnakoski et al. 2012). Our inoculation trials, conducted under field conditions, confirm that *O. clavatum* is phytopathogenic yet displays only low virulence toward its host. However, lesion severity on Scots pine seedlings in our experiments was greater than that reported in analogous studies performed under controlled conditions (Isberg et al. 2022), suggesting that environmental stressors may exacerbate fungal impact.

Lieutier et al. (2009) proposed that associated blue‐stain fungi facilitate beetle establishment on trees by stimulating and subsequently exhausting host defenses, thereby assisting their insect vectors in overcoming plant resistance mechanisms. The induction of defensive responses in Scots pine tissues following *O. clavatum* inoculation is evidenced by the accumulation of defensive compounds—such as phenols, terpenes, and lignin—within the reaction zone (Guérard et al. 2007; Villari 2012; Villari et al. 2012), as well as the upregulated expression of defensins, which are key components of innate plant immunity (Yusypovych et al. 2025).

The defensive system of Scots pine colonized by *Ips acuminatus* can be depleted not only by ophiostomatoid fungi but also by other members of the fungal community associated with the beetle, particularly pathogenic fungi (Davydenko et al. 2017). In the present study, an isolate of *F. verticillioides*, a member of the *Fusarium fujikuroi* species complex, was recovered from blue‐stained sapwood. This species is globally recognized as an aggressive pathogen responsible for *Fusarium* ear rot in maize and damping‐off diseases in various pine species, including *Pinus sylvestris* , *P. pinea* , and *P. nigra* (Deepa and Sreenivasa 2017; Olaizola et al. 2023). It has also been identified as the causal agent of resinous canker in *Pinus greggii* (De León‐Torres et al. 2023). The failure of inoculated seedlings to achieve wound closure, contrasted with the complete cambial healing in controls, indicates that *F. verticillioides* significantly impairs the host's defensive recovery. The depth of necrosis, reaching the second annual ring, indicates an ability to bypass lignosuberized boundary zones (Elfstrand et al. 2020).

In Ukraine, *I. acuminatus* and *I. sexdentatus* have previously been associated with *Fusarium* species such as *F. oxysporum* and *F. avenaceum* (Davydenko et al. 2017, 2021). In Spain, both *F. circinatum* and *F. verticillioides* have been detected in association with *I. sexdentatus* (Romón et al. 2008). The presence of *F. verticillioides* in sapwood raises questions regarding its entry pathways, possibly including beetle‐mediated transmission or environmental entry via the roots (Ren et al. 2024).

Our in vitro results revealed pronounced asymmetric competition, in which *F. verticillioides* acted as a highly aggressive competitor, reducing the growth of *O. clavatum* by approximately 45%, and eliciting morphological responses frequently associated with biotic stress (Fomina et al. 2024). Such strong competitive dominance in vitro suggests that, under natural conditions, *F. verticillioides* may establish dominance during the early stages of wood colonization, potentially limiting the spatial distribution of *O. clavatum*. Furthermore, the possibility of a synergistic interaction between these fungi cannot be excluded; co‐infection might enable them to overcome host defenses, such as lignosuberized barriers (Elfstrand et al. 2020), thereby exacerbating tree decline. This phenomenon could amplify mortality in Scots pine forests under the compounding stress of climate change, similar to complex symbioses observed in other bark beetle systems (Morales‐Ramos et al. 2000; Bayman and Serrato‐Diaz 2025).

## 5 Conclusions

This study provides the first evidence of *O. clavatum* associated with *I. acuminatus* in Ukraine, significantly expanding its documented geographic range in Europe and supporting the hypothesis of broad spatial consistency for this specific beetle–fungus association. Inoculation trials confirmed that while *O. clavatum* is phytopathogenic, it exhibits low virulence, likely contributing to host weakening rather than direct tree mortality. In contrast, *F. verticillioides* demonstrated strong pathogenicity on Scots pine seedlings and competitive dominance over *O. clavatum* in vitro, suggesting its potential role as a key contributor to pine decline.

Under the current trends of global climate change, characterized by prolonged droughts and thermal stress, these fungal‐beetle associations may exert increased pressure on pine ecosystems, further predisposing trees to infestation and accelerating forest dieback. While our study focused on identifying the dominant fungal drivers, we frame these findings as a valuable baseline for Western Ukraine. We emphasize the need for larger‐scale follow‐up studies—utilizing high‐throughput sequencing such as ITS metabarcoding and shotgun metagenomics—to fully characterize the broader community diversity and the functional dynamics of the core mycobiome. Such integrated approaches are essential to elucidate the multipartite interactions within bark beetle‐vectored fungal communities and their evolving role in tree susceptibility and forest decline.

## Funding

This work was supported by the National Research Foundation of Ukraine (2021.01/0184) and Simons Foundation (SFI‐PD‐Ukraine‐00014576).

## Conflicts of Interest

The authors declare no conflicts of interest.

## Supporting information


**Figure S1:** Sources of fungal isolates. (a) Scots pine logs infested with *Ips acuminatus*; (b) adult beetles and larvae of *I. acuminatus*.

**Table S1:** Strains used for phylogenetic analysis, their characteristics, and percent identity to isolate F3‐1.

## Data Availability

The data that support the findings of this study are openly available in NCBI GenBank at https://www.ncbi.nlm.nih.gov/genbank/, reference numbers OR799511.1, PX237223.1, PX237224.1, OR799512.1, PQ488808.1, PQ527913.1, PV976806.1, and PV976805.1. The data that supports the findings of this study are available in the Supporting Information of this article, and the additional data may be provided upon request to the corresponding author.
