# Supplementary material for: First Report of Ophiostoma clavatum and Fusarium verticillioides Associated With Ips acuminatus‐Infested Scots Pine in Western Ukraine
Source: Plant Environ Interact. 2026 Feb 23;7(2):e70134. doi: 10.1002/pei3.70134 (PMC12929033; doi:10.1002/pei3.70134)
Supplement: Supplementary file 1 — Figure S1: Sources of fungal isolates. (a) Scots pine logs infested with Ips acuminatus; (b) adult beetles and larvae of I. acuminatus. [file PEI3-7-e70134-s002.docx]

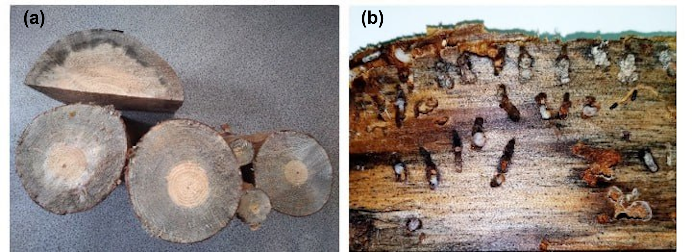


**Figure S1.** Sources of fungal isolates. (a) Scots pine logs infested with *Ips acuminatus*; (b) adult beetles and larvae of *I. acuminatus.*
